# Extraction and cleansing of data for a non-targeted analysis of high-resolution mass spectrometry data of wastewater

**DOI:** 10.1016/j.mex.2018.04.008

**Published:** 2018-04-17

**Authors:** Yaroslav Verkh, Marko Rozman, Mira Petrovic

**Affiliations:** a Catalan Institute for Water Research (ICRA), Carrer Emili Grahit 101, 17003 Girona, Spain; b Ruđer Bošković Institute, Bijenička cesta 54, 10000, Zagreb, Croatia; c Catalan Institution for Research and Advanced Studies (ICREA), Passeig Lluís Companys 23, 08010, Barcelona, Spain

**Keywords:** Combined MZmine 2.26 and R extraction workflow of LC-HRMS wastewater data, Water screening, Molecular formula prediction, R, MZmine

## Abstract

We provide a workflow to extract unidentified signals from chromatography-high resolution mass spectrometry (LC-HRMS) data of wastewater samples as a pre-step of a non-targeted analysis of dissolved organic matter (DOM). We provide detailed methodology on data processing and cleanup using MS processing software MZmine 2 and an own set of functions in R developed for wastewater analysis. The processing involves signal extraction, linear mass correction, reduction of noise, grouping of isotopologues, molecular formula assignment and merging of replicates. The article contains software settings and reasoning behind the choice of data extraction options. The supplementary information contains a script for the correction of signal masses using internal standards and templates of internal standard lists. We included a reproducible example as an R notebook with data cleansing workflow and data exported from MZmine. The data were used according to the described methodology in the article “A non-targeted high-resolution mass spectrometry data analysis of dissolved organic matter in wastewater treatment” by Verkh et al., 2018.

• Includes a linear mass correction algorithm for LC-HRMS signals.
• Describes a pipeline of non-targeted processing of LC-HRMS data of wastewater using free software.
• Provides tests and reasons for parameter choice in non-targeted LC-HRMS wastewater data extraction.

## Graphical abstract

**Figure:**
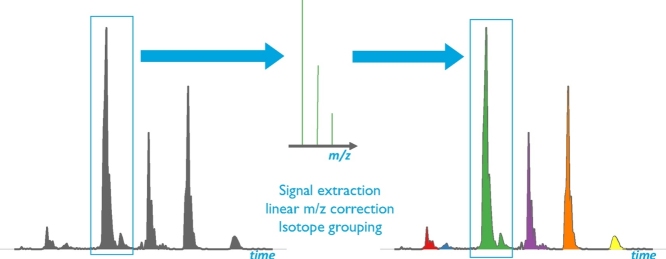

Specifications Table

**Table:** 

Subject area	Chemistry
More specific subject area	Environmental chemistry
Method name	Combined MZmine 2.26 and R extraction workflow of LC-HRMS wastewater data
Name and reference of original method	Not applicable
Resource availability	http://MZmine.github.io/ (MZmine user version)
	https://github.com/MZmine/MZmine2 (MZmine developer version)
	https://github.com/Tebatu/mzmine2 (MZmine customized developer version)
	https://bitbucket.org/Tebatu/MZminer

## Method details

### Extraction and cleaning up of data

The methodology was developed and tested with LC-HRMS data recorded on an Orbitrap Velos mass spectrometer. The measurement included filtered wastewater samples and blank in triplicate recorded at a resolution of 100 000 full width at half maximum. Details on the method and pretreatment can be found in [1]. LC-HRMS data of wastewater treatment samples were extracted with a custom version of MZmine 2.26 in which the atomic ratios in formula prediction were set to H/C < 3.2, O/C < 1.2, N/C < 1.3, S/C < 0.8. The formula prediction yielded output in form of “FormX IsoY MassZ”, where X is neutral formula, Y is isotopic pattern score between 0 and 1, and Z is neutral monoisotopic mass of the predicted formula in Da. Within MZmine pipeline, R was used to correct *m/z* of extracted signals using a linear model based on internal standards (IS). Exemplary IS lists for *m/z* correction in both ionization modes were stored in Supplementary Files with the required order and naming of table columns within files. The files in csv-format are readable in table format into spreadsheet editors as Microsoft Excel^®^ or OpenOffice Calc.

### MZmine 2.26 parameters in electrospray positive ionization (PI) mode

• Mass Detection: “Exact mass” detector with an intensity noise level of 1∙10^3^ a.u.
• FTMS Shoulder peaks filter: The Lorenzian extended peak model function with a mass resolution of 1∙10^5^.
• Chromatogram builder: A minimum time span of 0.03 min (2 s); a minimum signal height of 1∙10^3^ a.u.; a mass tolerance of extraction 5 ppm.
• Chromatogram smoothing: Filter width of 7 points.
• Chromatogram deconvolution: Noise amplitude algorithm was applied with a signal duration of 0.05–8.00 min, a minimal signal height of 3∙10^3^ a.u. and an amplitude of noise 1∙10^3^ a.u.
• Custom database search for IS: mass tolerance <5 ppm, retention time tolerance <0.3 min. A visual check of the IS signals confirmed their validity.
• A personal script in R corrected the *m/z* of signals in exported XML peak lists using linear models based on IS. The linear models were calculated for two *m/z* ranges over and under 400 Da. The script removed IS outliers >4* interquartile range and omitted the correction where less than 3 IS signals defined the model. Exemplary R script “mzCorrectionWithIs.R” and IS list “internal_standards_for_mz_correction_PImode.csv” that is used within can be found in Supplementary Files.
• Isotopic peaks grouper: 60 ppm mass tolerance, 0.03 min of time tolerance and a maximum charge of 2, representative peak: lowest *m/z* and monotonic shape.
• Filters: peaks in an isotope pattern ≥2, peak per row ≥1, points per peak 7–500.
• Adduct search: retention time tolerance <0.03 min, given a prevalent ionization [M+H]^+^ the adducts in PI LC-ESI-MS are: [M + NH_4_]^+^, [M + Na]^+^, [M + 2Na]^2+^, [M + K]^+^, [M + 2K]^2+^, [M + CH_3_OH]^+^, mass tolerance 5 ppm and maximum adduct signal height of 50%. Subsequently these adducts were removed from the feature list.
• RANSAC aligner: Mass tolerance 5 ppm, retention time tolerance 2.00 min, retention time tolerance after correction 1.00 min. Number of iterations was set by the software, 60% of points in the model were considered for validation, fit threshold of 1 min. A linear model was not assumed. The same charge was required.
• *m/z* and RT gap filler: mass tolerance of 10 ppm.
• List of aligned features was separated by charge to contain either charge 1 or 2.
• Duplicate peak filter: 3 ppm mass tolerance, 0.03 min retention time tolerance.
• Formula prediction: The ionization type was [M+H]^+^ and the mass tolerance of prediction 5 ppm. Atomic ranges used for prediction: C_1-80_, H_1-100_, O_0-20_, N_0-15_, S_0-4_, Cl_0-4_, Br_0-4_. Heuristic rules and RDBE restrictions of 0–40 RDBE were applied. A custom built MZmine version allowed the atomic ratios H/C < 3.2, O/C < 1.2, N/C < 1.3, S/C < 0.8. Isotopic pattern comparison enhanced the prediction with a mass tolerance of isotopes set to 5 ppm, a minimum absolute intensity of 6∙10^2^ a.u. and a minimum match score of 60%. Depending on the data, the charge varied between 1 and 2. Features with charge 2 and *m/z* > 500 Da did not receive an elemental formula. The custom-built formula prediction yielded output in form of “FormX IsoY MassZ”, where X is neutral formula, Y is isotopic pattern score between 0 and 1, and Z is neutral monoisotopic mass of the predicted formula in Da. The best formula candidate was picked in R using the output according to the algorithm described below.
• Export of csv file with *m/z*, retention time, and all IDs of features. Heights, areas, and charges of features were exported.

### MZmine 2.26 parameters in electrospray negative ionization (NI) mode

• Mass Detection: “Exact mass” detector with an intensity noise level of 2.5∙10^3^ a.u.
• FTMS Shoulder peaks filter: The Lorenzian extended peak model function with a mass resolution of 1∙10^5^.
• Chromatogram builder: A minimum time span of 0.03 min (2 s); a minimum signal height of 2.5∙10^3^ a.u.; a mass tolerance of extraction 5 ppm.
• Chromatogram smoothing: Filter width of 7 points.
• Chromatogram deconvolution: Noise amplitude algorithm was applied with a signal duration of 0.05–8.00 min, a minimal signal height of 7.5∙10^3^ a.u. and an amplitude of noise 2.5∙10^3^ a.u.
• Custom database search for IS: mass tolerance <5 ppm, retention time tolerance <0.3 min. A visual check of the IS signals confirmed their validity.
• A personal script in R corrected the *m/z* of signals in exported XML peak lists using linear models based on IS. The linear models were calculated for two *m/z* ranges >400 Da and <400 Da. The script removed IS outliers >4 * interquartile range and omitted the correction where less than 3 IS signals defined the model. Exemplary R script “mzCorrectionWithIs.R” and IS list “internal_standards_for_mz_correction_NImode.csv” that is used within can be found in Supplementary Files.
• Isotopic peaks grouper: 60 ppm mass tolerance, 0.03 min of time tolerance and a maximum charge of 2, representative peak: lowest *m/z* and monotonic shape.
• Filters: peaks in an isotope pattern ≥2, peak per row ≥1, points per peak 7–500.
• Adduct search: retention time tolerance 0.03 min, given a prevalent ionization [M−H]^−^ the adducts in NI LC-ESI-MS are : [M−H_2_O−H]^−^, [M−2(H_2_O)−H]^−^, [M-2H + K]^−^, [M−2H + Na]^−^, [M + Cl]^−^, [M−H + HAc]^−^, [M + Br]^−^, [M−H + FA]^−^, mass tolerance 5 ppm and maximum adduct signal height of 50%. Subsequently, the adducts were removed from the feature list.
• RANSAC aligner: Mass tolerance 5 ppm, retention time tolerance 2.00 min, retention time tolerance after correction 1.00 min. Number of iterations was set by the software, 80% of points in the model were considered for validation, fit threshold of 1 min. A linear model was not assumed. The same charge was required.
• *m/z* and RT gap filler: mass tolerance of 10 ppm.
• List of aligned features was separated by charge to contain either charge 1 or 2.
• Duplicate peak filter: 3 ppm mass tolerance, 0.03 min retention time tolerance.
• Formula prediction: The ionization type was [M+H]^−^ and the mass tolerance of prediction 5 ppm. Atomic ranges used for prediction: C_1-80_, H_1-100_, O_0-20_, N_0-15_, S_0-4_, Cl_0-4_, Br_0-4_. Heuristic rules and RDBE restrictions of 0–40 RDBE were applied. A custom built MZmine version allowed the atomic ratios H/C < 3.2, O/C < 1.2, N/C < 1.3, S/C < 0.08. Isotopic pattern comparison enhanced the prediction with a mass tolerance of isotopes set to 5 ppm, a minimum absolute intensity of 1∙10^3^ a.u. and a minimum match score of 60%. Depending on the data the charge varied between 1 and 2. Features with charge 2 and *m/z* > 500 Da did not receive an elemental formula. The custom-built formula prediction yielded output in form of “FormX IsoY MassZ”, where X is neutral formula, Y is isotopic pattern score between 0 and 1, and Z is neutral monoisotopic mass of the predicted formula in Da. The best formula candidate was picked in R using the output according to the algorithm described below.
• Export of csv file with *m/z*, retention time, and all IDs of features. Heights, areas, and charges of features were exported.

Picking of the best candidate for the molecular formula of the molecular feature, cleaning up of data and calculation of elemental properties were performed in R. The workflow examples are in R notebooks stored in Supplementary Files and contain functions from own R package [2]. The files “mmc3.csv” and “mmc4.csv” stored in Supplementary Files contain MzMine 2 raw output of real wastewater samples in PI and NI mode respectively, described in [1] in form of *m/z*, retention tme, potential elemental composition, their intensity, and charge for all processed samples. The files are readable in table format in Microsoft Excel®. The authors advise to overview the files upon import in R or another data processing environment, since the large amount of information may slow down the function of more visual editors. The files are to be used with R Studio notebook “MZmine_data_cleanup.Rmd” to show a reproducible example of the data cleaning up in R described below.

### Data cleaning up in R

• IS were removed from datasets.
• Picking the best candidate for the molecular formula: The candidate with the highest isotopic pattern score was selected unless the candidate with the next highest isotopic score had a deviation in isotopic score <10% and absolute ppm error <1 ppm compared to the first candidate. In that case, the algorithm picked the second candidate formula.
• Avoiding “square” peaks which are peak extraction artifacts in MZmine: Features with an area-to-height ratio >30 were removed. This value was chosen for this dataset through observation.
• Filtering by CV: Features with a CV >30% were removed.
• Baseline correction: Features with a sample-to-blank area ratio <3 were removed. Features with a sample-to-blank area ratio >3 were corrected by the area of the blank samples if the CV of the blank samples was <30%.
• Molecular features in datasets of PI and NI modes were combined and the variable neutral mass was calculated for features from *m/z*, considering the charge of features, the ionization mode and loss/gain of hydrogen.
• Duplicate removal: PI and NI features were united and the duplicates removed for the mass deviation < 5 ppm and retention time deviation <0.5 min for [M+H]+ in PI mode and for [M−H]− in NI mode under the assumption that the retention time is similar in both PI and NI modes because both were recorded under the same chromatographic method.
• The elemental properties as the number of atoms in a molecule, atomic ratios of type X/C (where X is H, N, O, S), double bond equivalents (DBE), and DBE minus count of O atoms in the molecule (DBE-O) were calculated using the neutral molecular formula of a molecular feature, if a molecular formula was assigned. DBE was calculated using the equation DBE = C + 1 + (N – X −H)/2 where X is the sum of halogen atoms in a molecule and DBE-O was calculated by subtracting the number of O atoms in a molecule from DBE.

### Custom MZmine build

To install the developer version of MZmine follow instructions on http://mzmine.github.io/eclipse_tutorial.html. The custom build of MZmine used in the publication can be downloaded from https://github.com/Tebatu/mzmine2. Alternatively, it can be built by modifying the original developer version. For this in file FormulaPredictionPeakListTask.java change lines

**Figure:**
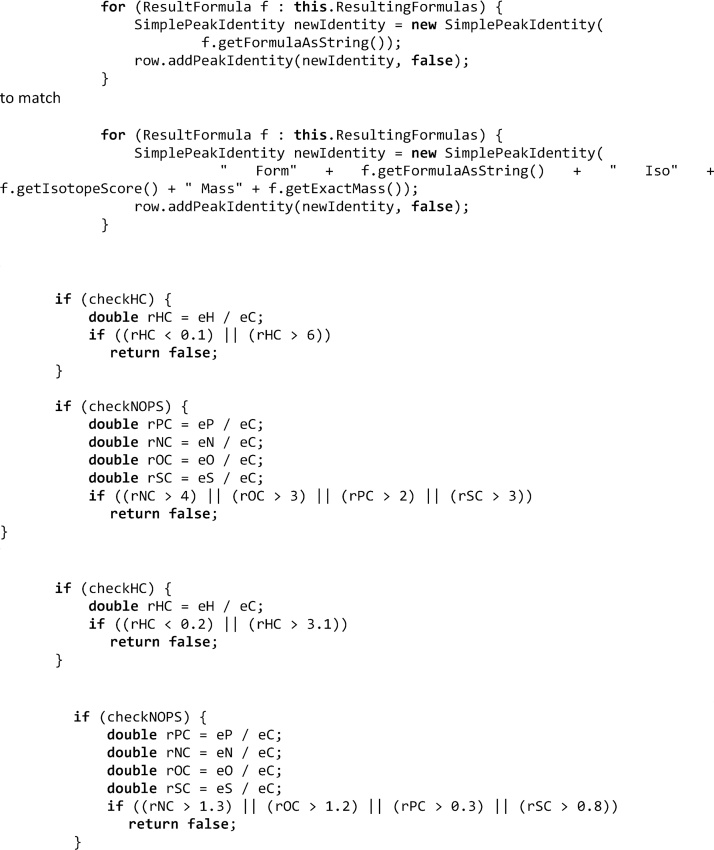

## Method validation

Signals with signal-to-baseline <3 were ignored in MZmine to exclude instrumental noise. Orbitrap–MS is a Fourier transform based technique and signals show satellites that arise during the conversion of current to spectral data. These satellite signals were ignored under the assumption of 1∙10^5^ mass resolution in the measurement to ensure exclusion of noise and faster computation. The mass tolerance for the processing of LC-HRMS spectra was set to 5 ppm. The mass error is <2 ppm for proxies in these data. But, the precision falls increasingly with the loss of intensity, so the established threshold of 5 ppm is more realistic considering the literature suggestions about the negative impact of too stringent tolerance margins [3,4].

Durations of the extracted signals were between 0.05 and 8.00 min. The peak duration was focused on extracting all signals in the spectra while controlling for the column bleed considering the applied chromatographic method.

A mathematical baseline correction was not applied, to avoid an uncontrolled loss of information. The shown data was corrected by subtracting signal intensity of solvent blank from the matching signal in a sample to apply correction only to relevant chromatographic regions.

Only monoisotopic signals with at least one consecutive isotopologue were analyzed. Grouping of isotopes into a feature represented by one monoisotopic mass reduces the data stream and adds isotopic information to the features. Ungrouped signals were removed to makes sure that only molecular features with an isotopic pattern are used for the formula prediction. The choice to use a 60 ppm window in isotopic grouping is explained by the way MZmine picks isotopologues. The software performs well for natural organics as carbohydrates since it groups isotopes by assuming the default distance of ∼1.0033 Da. The matching of isotopic pattern turns out to be increasingly difficult for molecules with a strong mass defect, for example, Cl-containing molecules, since the isotopologues seem to deviate beyond the threshold <5 ppm. Using a set of known compounds we found 60 ppm to be a value at which we do not have grouping of random signals of isotopic patterns, but at the same time, (while using a monotonic shape of isotopic pattern as a setting) we get a very good recognition of the pattern for molecules with strong mass defects. Only protonated or deprotonated ions with a charge ± 1 or ±2 were analyzed. ESI is a soft ionization method and wastewater DOM largely consists of small molecules, which cannot accumulate many charges. The IS showed a prevalent [M+H] + in PI and [M-H]- in NI modes.

Search for adducts further reduced the dataset. The intensity of adduct features was not added to the intensity of the primary molecular features since it was shown that this does not offer a significant improvement of data [5]. Molecular features that corresponded to adducts [M + NH_4_]^+^, [M + Na]^+^, [M + 2Na]^2+^, [M + K]^+^, [M + 2 K]^2+^, [M + CH_3_OH]^+^ in PI mode and [M−H_2_O−H]^−^, [M−2(H_2_O)−H]^−^, [M−2H + K]^−^, [M−2H + Na]^−^, [M + Cl]^−^, [M−H+HAc]^−^, [M + Br]^−^, [M−H + FA]^−^ in NI mode were removed in MZmine.

A correction of the ion suppression by matrix using an intensity normalization of spectra was not attempted. A normalization leads to worse results in replicates compared to the unaltered spectra. The influence of the matrix effect can be reduced by standardizing signal intensity [6]. The automatic standardization requires a set of IS throughout the entire spectrum. The amount of IS in the study did not cover the range and not enough signals had the required high intensity to correctly normalize the spectra. CV of features among the replicates of a sample has to decrease after a successful standardization while here the CV increased.

Molecular formulae were predicted for molecular features and, where discovered, assigned. The prediction was only allowed for features with neutral mass <1000 Da to prevent a high rate of false assignments for heavy molecules. the atomic ratios of generated formulae were H/C < 3.2, O/C < 1.2, N/C < 1.3, and S/C < 0.8 which corresponds to 99.7% of registered small molecules [7]. The presented workflow prioritized the isotopic pattern score to select the best candidate for the molecular formula.

The ranges for the DOM elements CHONPS, which are common for organic matter, and halogens FClBrI that can occur in synthetic organics, were derived from 14,631 micro-contaminants [8]. These helped to establish suitable parameters for prediction of molecular formula for DOM and in particular for organic micro-contaminants. Common elements C, H, O, and N have narrow distributions and were included in the prediction ranges at values approaching their maxima. S, P, Cl, Br, F and I have a median close to zero and many outliers. S, Br, and Cl are important in the wastewater treatment and they were included in the formula prediction at sensibly low values.

A balanced atomic range excluded a statistical appearance of wrong formulae, but contained enough atoms for most formulae to be predicted, when F and P are excluded. F and P are problematic for the prediction because they do not carry any kind of isotopic information. This leads to a false assignment of F and P to substances that contain none. The applicability of formula prediction was tested using experimental data of pharmaceuticals in PI and NI mode, which covered major composition-relevant atomic properties of micro-pollutants. The samples with pharmaceuticals underwent the same signal detection, *m/z* correction, isotope grouping, and formula prediction as the wastewater samples in this study. A subsequent targeted screening for [M+H]^+^ with an *m/z* error of 5 ppm was used to identify the molecular features of the pharmaceuticals within the non-targeted data. A correct prediction was challenging for molecules with high masses and with a low signal intensity. Pharmaceuticals that contained F could not receive a correct formula under the applied settings. Overall, 69 out of 79 formulas were predicted correctly using the workflow.

A gap-filling algorithm within MZmine looked for signals in the feature list of combined samples which were potentially missed during peak extraction. The algorithm reconstructs omitted chromatographic peaks for a molecular feature in “empty” samples by scanning the *m/z* and retention time region of LC–MS spectra corresponding to the detected peaks in other samples. The methodology later avoids noise recognized as peaks to enter the final list of molecular features by removing samples with CV-threshold >30% intensity deviation.

Some features in the dataset showed duplicates which appear during peak recognition and alignment of samples. The duplicates falsify the exploratory analysis of data and were removed in MZmine. To avoid removal of false duplicates from the dataset the *m/z* and retention time tolerances were set smaller than in previous MZmine modules to 3 ppm and 0.03 min respectively.

An R function was used to reduce random noise in the MZmine output data by removing samples with a CV of triplicate intensity >30%. CV of feature intensity is <30% threshold independent of the sample matrix. The 2 outliers out of 32 IS were ronidazole-d3 and simvastatin-d6. The feature intensity was corrected by subtracting the mean intensity of solvent blank from the sample, but only for signals with an intensity RatioSample:Blank >3. Signals with RatioSample:Blank <3 were ignored. This approach accounts for intensity deviations caused by the matrix suppression [9].
